# Centenarians Alleviate Inflammaging by Changing the Ratio and Secretory Phenotypes of T Helper 17 and Regulatory T Cells

**DOI:** 10.3389/fphar.2022.877709

**Published:** 2022-06-02

**Authors:** Lixing Zhou, Meiling Ge, Yan Zhang, Xiaochu Wu, Mi Leng, Chunmei Gan, Yi Mou, Jiao Zhou, C Alexander Valencia, Qiukui Hao, Bin Zhu, Biao Dong, Birong Dong

**Affiliations:** ^1^ National Clinical Research Center for Geriatrics and Department of Geriatrics, West China Hospital, Sichuan University, Chengdu, China; ^2^ State Key Laboratory of Biotherapy, West China Hospital, Sichuan University, Chengdu, China; ^3^ Geroscience and Chronic Disease Department, The 8th Municipal Hospital for the People, Chengdu, China; ^4^ Interpath Laboratory, Pendleton, OR, United States; ^5^ Department of Preclinical Education, Lake Erie College of Osteopathic Medicine, Erie, PA, United States

**Keywords:** centenarians, inflammaging, T helper 17 cell, regulatory T cell, aging

## Abstract

The immune system of centenarians remains active and young to prevent cancer and infections. Aging is associated with inflammaging, a persistent low-grade inflammatory state in which CD4^+^ T cells play a role. However, there are few studies that have been done on the CD4^+^ T cell subsets in centenarians. Herein, the changes in CD4^+^ T cell subsets were investigated in centenarians. It was found that with aging, the old adults had higher levels of proinflammatory cytokines and lower levels of anti-inflammatory cytokines in plasma. The levels of CRP, IL-12, TNF-α, IFN-γ, IL-6 and IL-10 were further increased in centenarians compared to old adults. While the levels of IL-17A, IL-1β, IL-23 and TGF-β in centenarians were closer to those in young adults. The total CD4^+^, CD8^+^, Th17 and Treg cells from peripheral blood mononuclear cells (PBMCs) were similar among the three groups. It was observed that the ratio of Th17/Treg cells was elevated in old adults compared to young adults. The ratio was not further elevated in centenarians but rather decreased. In addition, the *ex vivo* PBMCs differentiation assay showed that increased Th17 cells in centenarians tended to secrete fewer proinflammatory cytokines, while decreased Treg cells in centenarians were prone to secrete more anti-inflammatory cytokines. These observations suggested centenarians alleviated inflammaging by decreasing the ratio of Th17/Treg cells and changing them into anti-inflammatory secretory phenotypes, which provided a novel mechanism for anti-aging research.

## Introduction

### Background

Centenarians are rare individuals who reach the age of 100 years, but this population is now the fastest growing sector in many countries. In 2015, there were an estimated 450,000 centenarians worldwide, and this number is expected to increase 8-fold to approximately 3.7 million by 2050. A notable feature of centenarians is an increase in healthy lifespan. Even after the age of 100, they still maintain relatively high cognitive function and physical independence and are highly resistant to lethal diseases such as stroke, cancer and cardiovascular diseases (Claesson et al., 2012; Pavlidis et al., 2012; Yasumichi et al., 2014; Emily and schoenhofen, 2016; Young and Kroczek, 2019). Due to their ability to delay or even prevent the occurrence of age-related diseases, many centenarians can spend almost their entire lives in good health (Perls, 2012). Therefore, centenarians can be regarded as good models of successful aging, and understanding the longevity mechanism in centenarians would benefit the superaging societies (Hashimoto et al., 2019). Current research has shown that healthy longevity is the result of the joint forces of genetic variants, social, behavioral factors and living environments. However, how and to what extent these factors individually, jointly and interactively affect the lifespan and healthspan of centenarians remain to be defined (Poon and Cheung, 2012). Although there have been a number of studies exploring these questions, most of these have only observed the relationship between genetic, social, behavioral factors and longevity and have not conducted in-depth mechanistic studies on these factors (Zeng et al., 2017).

Aging is accompanied by restructuring changes in the immune system, which are collectively designated as “immunosenescence” (Nikolich-Žugich, 2018; Duggal et al., 2019; Goronzy and Weyand, 2019; Borgoni et al., 2021). One of the halls marks of immunosenescence is inflammaging, which is a persistent low-grade inflammatory state that accompanies aging (Aiello et al., 2019) and is characterized by elevated levels of blood pro-inflammatory factors such as C-reactive protein (CRP), tumor necrosis factor (TNF)-α and interleukin (IL)-6 (Franceschi et al., 2017). Inflammaging occurs in most older adults, and it has a high susceptibility to age-related morbidity and mortality (Ferrucci and Fabbri, 2018; Furman et al., 2019). One of the potential mechanisms of inflammaging is immune cell dysregulation (Ferrucci and Fabbri, 2018), manifested by increased inflammatory cytokines and changes in T cells. The adaptive immune response is now considered to be the immune response most severely affected by aging and is characterized by alterations in T cell phenotypes and functions (Fülöp et al., 2013). These changes form a low-grade inflammatory state in which CD4^+^ T cells play an important role (Schmitt et al., 2013). Senescent T cells secrete abundant inflammatory cytokines and mediators such as IL-6 and CRP (Aiello et al., 2019; Mittelbrunn and Kroemer, 2021). Studies have found that inflammation levels are closely related to longevity and can predict successful aging at an extremely old age (Arai et al., 2015). But among the many inflammation-related factors, only IL-6, TNF-α, and CRP levels were assessed in that study. Other studies have mainly focused on those younger than 100 years old, and only a few studies have been performed on the levels of various inflammation-related factors in centenarians.

The level of inflammatory cytokines in inflammaging is associated with reduced functionality and altered distribution of immune cells, among which age-related changes in T cells play an important role (Mittelbrunn and Kroemer, 2021). T cells mainly include CD4^+^ and CD8^+^ T cell populations. CD4^+^ T cells are helper cells that regulate the function of all the other immune cells. They also have effector functions (Das et al., 2017). According to different functions, CD4^+^ T cells can be subdivided into T helper (Th)1, Th2, Th17, and regulatory T cell (Treg) subgroups (Golubovskaya and Wu, 2016), which are differentiated from common naive CD4^+^ T cells. However, most studies are limited to the total CD4^+^ T cell pool, with only a few studies conducted with Th1 and Th2 CD4^+^ T cell subsets, and even less is known about the impact of aging in centenarians on other lineages, such as Th17 cells and Tregs.

Herein, we determined the levels of different subsets of T cells and the related cytokine levels as well as unique hallmarks in centenarians that may characterize healthy aging.

## Materials and Methods

### Subjects

The research protocol was approved by the Research Ethics Committee of Sichuan University. Briefly, 218 community-dwelling centenarians (age ≥100 years), 104 old adults (60–79 years old) and 16 young (20–45 years old) healthy counterparts were recruited. Informed consent was provided by each participant or their proxy respondents before participating in the study. Data were collected through in-person one-to-one interviews and physical examinations.

### Blood Sample Measurements

Fasting venous blood samples were drawn in the morning. Blood collection and processing were performed under standardized conditions according to subsequent experiments. Routine blood tests and biochemical parameters were detected by a chemistry analyzer (Olympus AU400, Tokyo, Japan) and a hematology analyzer (MEDONIC CA620, Spånga, Sweden), respectively. For the flow cytometry (FCM) assay and differentiation experiments *in vitro*, plasma and human peripheral blood mononuclear cells (PBMCs) were obtained from heparinized blood samples using a lymphocyte separation medium.

### CD4^+^ T Cells Culture and Differentiation

CD4^+^ T cells were cultured and differentiated as previously described (Zhou et al., 2018). Briefly, magnetic beads were used to isolate naive CD4^+^ T cells from human PBMCs *ex vivo.* Cells were cultured in AIM-V medium with 10% fetal bovine serum. For Th17 cell differentiation, naive CD4^+^ T cells were stimulated on plates precoated with 10 μg/ml anti-CD3 and 4 μg/ml anti-CD28 and generated by culture under the following conditions: 40 ng/ml IL-6 and 3 ng/ml transforming growth factor β (TGF-β). For Treg cell differentiation, naive CD4^+^ T cells were stimulated on coated plates with 0.5 μg/ml anti-CD3 and 1 μg/ml anti-CD28, and the culture was grown in the presence of 10 ng/ml TGF-β and 10 ng/ml IL-2. The control group was precoated with PBS and cultured without IL-6, TGF-β, or IL-2.

### FCM and Enzyme-Linked Immunosorbent Assay

For FCM analysis, 1 × 10^6^ cells per sample were used, and the cells were labeled with CD4-BB515. After permeabilization and fixation, Th17 and Treg cells were incubated with IL-17A-BV421 or Foxp3-PE, respectively. For Th17 cells, the cells were stimulated for 4–6 h in an incubator (37°C, 5% CO_2_) with a leukocyte activation cocktail prior to antibody incubation. The cells were detected by FLow cytometry (BD LSRFortess, Franklin Lakes, NJ, United States) and analyzed utilizing the FlowJo software (Tree Star, Inc. San Carlos, CA, United States). To detect CD3, CD8, Th1 and Th2 cells, CD3-APC-Cy7, CD8-APC, interferon (IFN)-γ-PE, and IL-4-APC from BD Bioscience were used.

Cytokines in serum or culture supernatants were measured using a commercially available ELISA kit (eBioscience, San Diego, CA, United States) according to the manufacturer’s protocols.

### Real-Time PCR analysis

Total RNA was extracted from cells using TRIzol Reagent (Thermo Fisher Scientific, Carlsbad, CA, United States) following the manufacturer’s instructions. qPCR was performed using SYBR Green PCR Master Mix (Applied Biosystems, Carlsbad, CA, United States) with primers that amplified the following genes: ROR-γt (forward, 5′-CTG​CTG​AGA​AGG​ACA​GGG​AG-3'; reverse, 5′-AGT​TCT​GCT​GAC​GGG​TGC-3′), Foxp3 (forward, 5′- GAG​AAG​GAG​AAG​CTG​AGT​GCC​AT-3'; reverse, 5′- AGC​AGG​AGC​CCT​TGT​CGG​AT-3′), T-bet (forward, 5′-AAC​ACA​GGA​GCG​CAC​TGG​AT-3'; reverse, 5′- TCT​GGC​TCT​CCG​TCG​TTC​A-3′), GATA-3 (forward, 5′-ACC​GGC​TTC​GGA​TGC​AA-3'; reverse, 5′-TGC​TCT​CCT​GGC​TGC​AGA​C-3′) and GAPDH (forward, 5′- ACC​ACA​GTC​CAT​GCC​ATC​AC-3'; reverse, 5′-TCC​ACC​ACC​CTG​TTG​CTG​TA-3′). The final volume of each PCR was 20 µl. The PCR conditions were as follows: 94°C for 3 min, 35 cycles of 94°C for 30 s, 30 s at 58°C, and 1 min at 72°C. Analysis of the melting curves confirmed that the fluorescence signal originated from specific PCR products and not from primer dimers or other artifacts.

### Common Reagents and Antibodies

For T cell cultures, Round Bottom 96 Well TC-Treated Microplate (3,799, Corning, Corning, NY, United States), lymphocyte separation medium (LTS1077, TBD Bioscience, Tianjin, China), human naive CD4^+^ T cell Isolation Kit (130-094-131, Miltenyi Biotec, Cologne, Germany), AIM-V medium (0870112DK, Invitrogen, Carlsbad, NY, United States) and fetal bovine serum (Invitrogen) were used. Anti-human CD3 (85-16-0289-81) and anti-human CD28 (85-16-0037-81) antibodies were purchased from eBioscience. Human IL-2 protein (200-02-10) and human IL-6 protein (200-06-5) were obtained from PeproTech (Rocky Hill, NJ, United States). Recombinant human TGF-beta 1 protein (240-B-002) was purchased from R&D (Minneapolis, MN, United States). For FCM, the following reagents were used: Foxp3/Transcription Factor Staining Buffer Set (00-5523-00) was purchased from eBioscience. The Cytofix/Cytoperm™ Fixation/Permeabilization Solution Kit (554714), Leukocyte Activation Cocktail with BD GolgiPlug (550583), anti-human CD4 BB515 (564419), anti-human CD8 APC (555369), anti-human CD3 APC-cy7 (557832), anti-human Foxp3 PE (560046), anti-human IL-4 APC (554486), anti-human IFN-γ PE (559327), and anti-human IL-17 BV421 (562933) were obtained from BD Bioscience, Franklin Lakes, NJ, United States.

### Statistical Analysis

All analyses were performed using GraphPad Prism version 8.0 (La Jolla, CA, United States). If samples followed a Gaussian distribution, one-way analysis of variance (ANOVA) was used to compare the differences in outcomes between groups. When samples did not pass the normality test, a nonparametric test (Mann–Whitney test) was applied. All data were presented as the mean ± SEM. *p* < 0.05 was considered to indicate statistical significance.

## Results

### Characteristics of Centenarians

Overall, 218 centenarians, including 55 males and 163 females, were enrolled in this study. First, the general characteristics of centenarians were analyzed. The mean age of the group was 102.2 years. As expected, centenarians were more likely to be females (74.8%), and only 7.3% of participants were current smokers. For chronic diseases, the prevalence of hypertension, myocardial disease, diabetes mellitus, cerebrovascular disease, Parkinson’s disease, Alzheimer’s disease, respiratory disease, chronic kidney disease, tumors and osteoarthritis was 15.4, 7.8, 2.8, 4.1, 0.5, 10.6, 9.2, 1.8, 2.8 and 3.2%, respectively. Activities of daily living (ADL) scores and falls in the previous 12 months were classic indicators of physical function in elderly adults. The average ADL scores and falls for centenarians were 77.6 ± 22.3 and 1.6 ± 2.2, respectively. For the basic body measurements, the average values of body mass index (BMI), systolic pressures, diastolic pressures and heart rate were 21.8 ± 6.8 kg/m^2^, 144.4 ± 21.4 mmHg, 81.9 ± 14.8 mmHg and 61.1 ± 30.8 bpm, respectively. There were no sex differences across chronic diseases, ADL scores, the number of falls in the previous 12 months, BMI, blood pressure or heart rate. An overview of these data was given in Supplementary Table S1. Compared to the West China Health and Aging Trend (WCHAT) cohort from the same region (Wyz et al., 2020), with a mean age of 62.1 years, centenarians had a lower prevalence of chronic diseases, suggesting that centenarians have better health status than middle-aged adults (data not shown). This finding was consistent with previous reports that centenarians maintained relatively high levels of cognitive function and physical independence even compared with middle-aged adults (Yasumichi et al., 2014; Emily and schoenhofen, 2016), indicating that they were resistant to aging.

Routine blood tests and blood biochemical examinations were conducted. It was found that there was no significant difference in most hematology and blood biochemical tests between males and females, except for platelet (PLT), prealbumin (PA), estimated glomerular filtration rate (eGFR), creatinine (CREA), uric acid (UA), urea and kalium (K) (Supplementary Tables S2, S3).

### Centenarians Have Unique Levels of Inflammation-Related Factors

**FIGURE 1:**
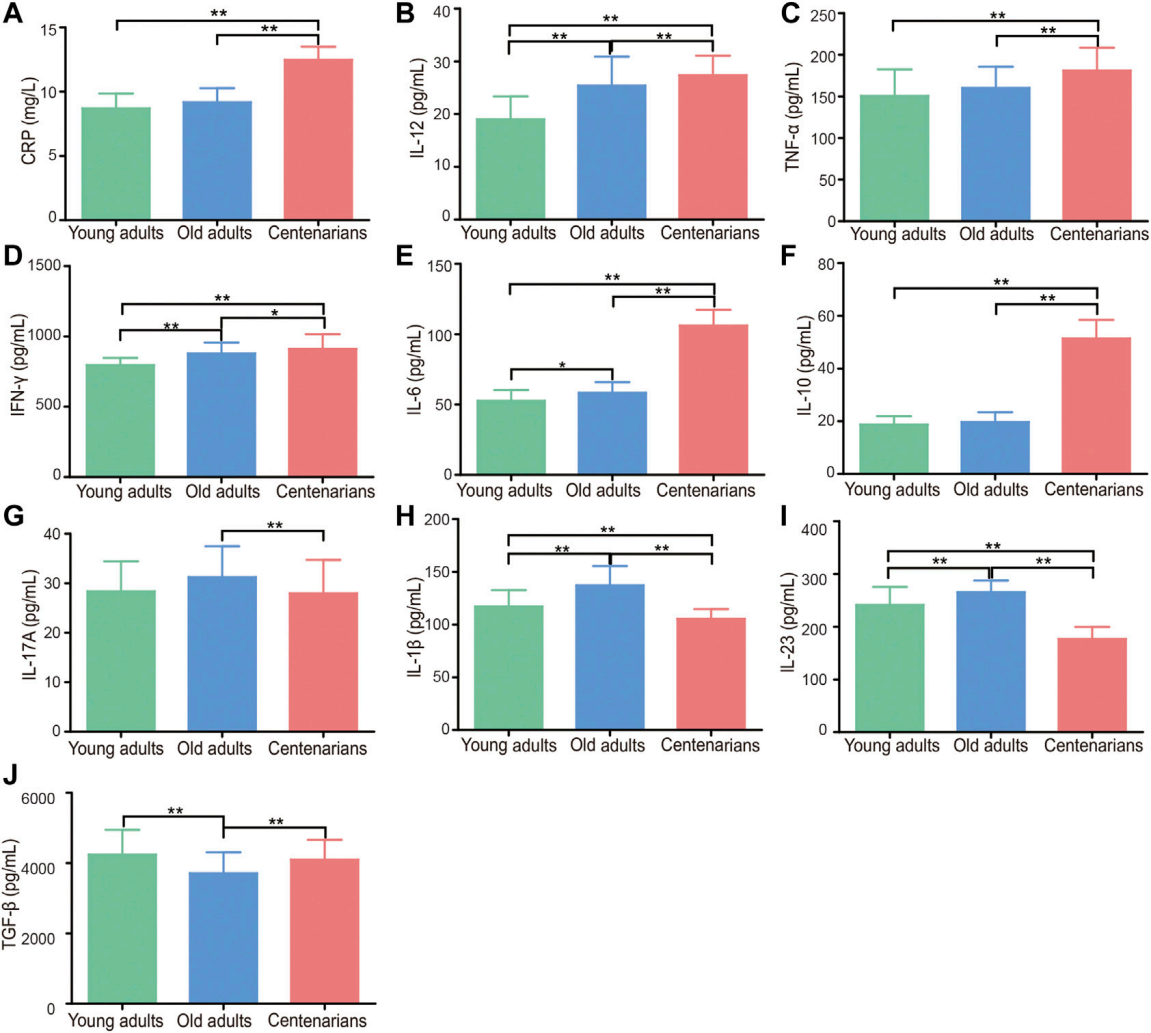
Centenarians have unique levels of inflammation-related factors. Inflammatory cytokines and mediators in human plasma, including CRP **(A)**, IL-12 **(B)**, TNF-α **(C)**, IFN-γ **(D)**, IL-6 **(E)**, IL-10 **(F)**, IL-17A **(G)**, IL-1β **(H)**, IL-23 **(I)** and TGF-β **(J)**, were detected by ELISA. n = 16, 104 or 218, respectively. Data were expressed as the mean ± SD. Asterisks (*) indicate significant differences (*p < 0.05; **p < 0.01).

According to previous studies (Baeza et al., 2011; Fülöp et al., 2013; Duggal et al., 2019; Goronzy and Weyand, 2019), elderly individuals have a state of immunosenescence, with high levels of inflammatory cytokines. Thus, we wondered whether centenarians as the oldest old have the highest levels of inflammation-related factors as important inflammaging hallmarks. Common inflammatory cytokines and mediators were measured in plasma to find evidence for this hypothesis. The characteristics of the study population were shown in Table 1. It was found that, compared with those in the plasma of young adults group, inflammation-related factors, including CRP, IL-12, TNF-α, IFN-γ, and IL-6, in the plasma of old adults and centenarians were increased, and they were positively correlated with age (Figures 1A–E). Interestingly, the anti-inflammatory cytokine IL-10 also showed a similar trend (Figure 1F). Intriguingly, some cytokines exhibited different patterns from the above cytokines in these groups. Among them, pro-inflammatory cytokines, including IL-17A, IL-1β, and IL-23, were increased, and the anti-inflammatory cytokine TGF-β was reduced in the old adults (Figures 1G–J). However, these cytokines in centenarians were closer to those in the young adults group, which is unexpected. The levels of these factors in different genders were also analyzed and no significant differences were found (data not shown). The above results indicated that centenarians had some degree of inflammaging, but the expression levels of some inflammatory cytokines were closer to those of young adults, which suggested that centenarians alleviated inflammaging compared with old adults.

**TABLE 1:** Characteristics of the study population.

Characteristics	Young Adults	Old Adults	Centenarians	*p* Value
N	16	104	218	
Age (χ±SEM)	28.6 ± 1.6	69.1 ± 0.6	102.2 ± 0.1	*p* < 0.01
Sex				
Male (%)	4 (25.0)	26 (25.0)	55 (25.2)	>0.05
Female (%)	12 (75.0)	78 (75.0)	163 (74.8)	>0.05

Groups were compared by nonparametric analyses.

### The Th17/Treg Cell Ratio Decreased in the Centenarians.

**FIGURE 2:**
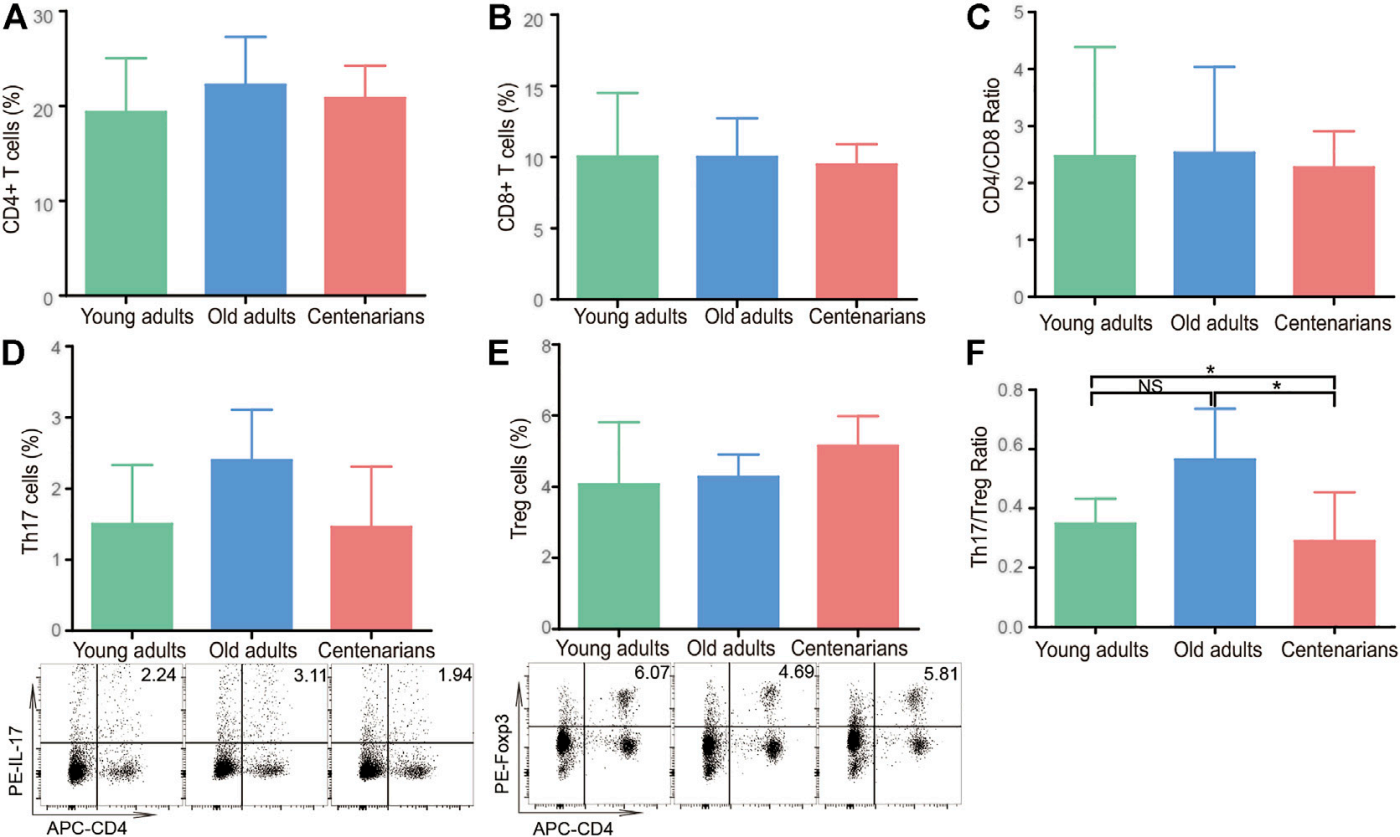
The Th17/Treg cell ratio decreased in centenarians. PBMCs were extracted from young adults, old adults and centenarians. CD4^+^ T cells **(A)**, CD8^+^ T cells **(B)**, and the ratio of CD4^+^/CD8^+^ cells **(C)** were analyzed by FCM. Th17 cells **(D)** and Treg cells **(E)** and the ratio of Th17/Treg cells **(F)** were analyzed. Data were expressed as the mean ± SD. n = 8. Asterisks (*) indicate significant differences (*p < 0.05; **p < 0.01); NS, not significant.

Since IL-17A, IL-1β, IL-23, and TGF-β are mainly secreted by CD4^+^ T cells (Duggal et al., 2019), the levels of T cells and their subsets in PBMC were investigated in centenarians. It was found that there was no significant difference between CD4^+^ T cells, CD8^+^ T cells, or the CD4/CD8 ratio in the different age groups (Figures 2A–C). CD4^+^ T cells mainly consist of Th1, Th2, Th17, and Treg cells, all of which can secrete cytokines (Goronzy and Weyand, 2019). Therefore, these 4 cell subsets were investigated next. It was observed that there were no significant differences in Th1 cells among the three groups as well as the mRNA levels of the transcription factor T-bet in Th1 cells (Supplementary Figure S1A,B). Similarly, the levels of Th2 cells and the related transcription factor GATA-3 did not differ between the three groups (Supplementary Figure S1C,D). In addition, the Th1/Th2 ratio levels did not change significantly with age (Supplementary Figure S1E). As for Th17 and Treg cells, there was an increasing Th17 cell trend in old adults compared to the young adults group. The Th17 cells in the centenarian group did not continue to rise but instead decreased compared to those in the old adults group, but this decrease was not statistically significant (Figure 2D). What’s more, there was no significant difference in Treg cells among the three groups (Figure 2E). Interestingly, the ratio of Th17/Treg cells showed a clear trend in which the ratio was elevated in the old adults group compared with the young adults group. However, the ratio in centenarians was significantly lower than that in the old adults group and even lower than that in the young adults group (Figure 2F). The above data suggested that the changes in cytokines in centenarians may be due to the decrease in the Th17/Treg ratio.

### CD4^+^ T Cells Derived From Centenarians Have Anti-Inflammatory Secretory Phenotypes.

**FIGURE 3:**
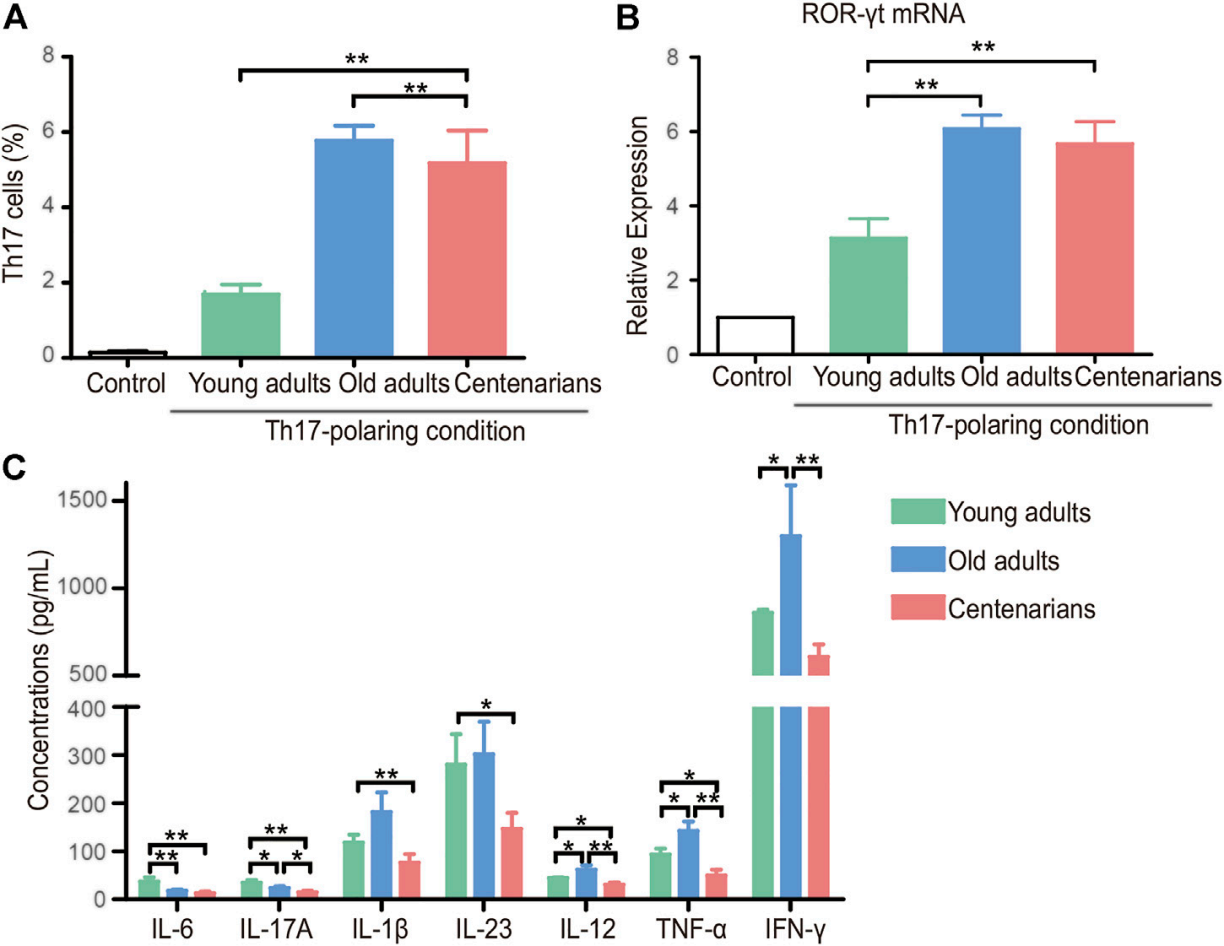
Increased Th17 cells in centenarians secreted fewer proinflammatory cytokines *in vitro*. Naive CD4^+^ T cells from young adults, old adults and centenarians were stimulated with immobilized anti-CD3 and anti-CD28 monoclonal antibodies under Th17-polarizing conditions for 5 days *in vitro*. **(A)** Th17 cells were measured by FCM. **(B)** ROR-γt mRNA levels were determined by qPCR in Th17-polarizing conditions. **(C)** The supernatants in Th17-polarizing conditions were collected to detect the levels of IL-6, IL-17A, IL-1β, IL-23, IL-12, TNF-α and IFN-γ by ELISA. Data were expressed as the mean ± SD. n = 8. Asterisks (*) indicate significant differences (*p < 0.05; **p < 0.01).

To explore what changes occurred in the T cells of centenarians, naive CD4^+^ T cells were extracted from people of different ages and differentiated under Th17- or Treg-polarizing conditions *in vitro*. We found a strong tendency of naive CD4^+^ T cells to differentiate into Th17 cells with increasing age under Th17-polarizing conditions. The Th17 cells in the old adults group were nearly three times higher than those in the young adults group, but the number in the centenarian group did not increase further (Figure 3A). The same trend was observed in the mRNA level of the transcription factor of Th17 cells, retinoid-related orphan nuclear receptor (ROR)-γt (Figure 3B). Moreover, Th17-related proinflammatory cytokines, including IL-6, IL-17A, IL-1β, IL-23, IL-12, TNF-α, and IFN-γ, were observed in cell culture supernatants under Th17-polarizing conditions. It was seen that as the differentiation of Th17 cells increased, the levels of most cytokines in the old adults group were elevated compared to those in the young adults group. Intriguingly, in the centenarian group, the levels of these cytokines were significantly lower than those in the old adults groups and even lower than those in the young adults group (Figure 3C).

**FIGURE 4:**
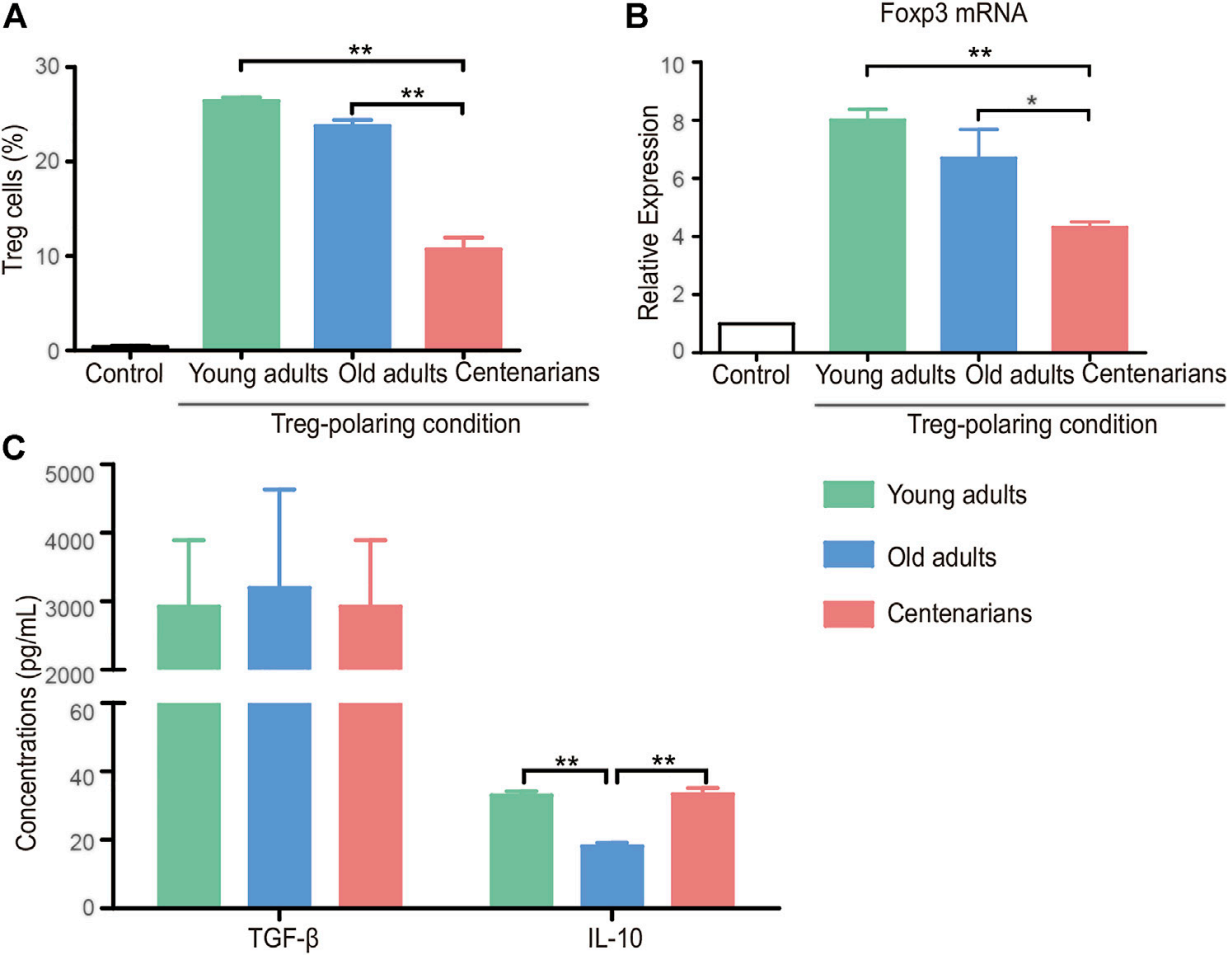
Decreased Treg cells in centenarians secreted more anti-inflammatory cytokines *in vitro*. Naive CD4^+^ T cells from young adults, old adults and centenarians were stimulated with immobilized anti-CD3 and anti-CD28 monoclonal antibodies under Treg-polarizing conditions for 5 days *in vitro*. **(A)** Treg cells were measured by FCM. **(B)** Foxp3 mRNA levels were determined by qPCR in Treg-polarizing conditions. **(C)** The supernatants in Treg-polarizing conditions were collected to detect the levels of TGF-β and IL-10 by ELISA. Data were expressed as the mean ± SD. n = 8. Asterisks (*) indicate significant differences (*p < 0.05; **p < 0.01).

Under Treg-polarizing conditions, the proportion of naive CD4^+^ T cells that differentiated into Treg cells decreased with age in both the old adults group and the centenarian group compared with the young adults group, and there was a further reduction of Treg in the centenarians compared with that in the old adults. (Figure 4A). A similar trend of Forkhead box protein P3 (Foxp3) as the transcription factor of Treg was seen in the three groups (Figure 4B). Subsequently, the detection of the relevant anti-inflammatory cytokines in the Treg-polarizing cell culture supernatants revealed that although the number of Treg cells was significantly lower in the centenarian group than in the young adults group, the level of TGF-β was not significantly lower, and the levels of IL-10 were higher compared with those secreted by the old adults group (Figure 4C), suggesting that individual Treg in centenarians secretes more anti-inflammatory cytokines than in old adults.

Altogether, the results suggested that naive CD4^+^ T cells derived from centenarians were more likely to differentiate into Th17 cells and less likely to differentiate into Treg cells *in vitro.* However, the secretory function of Th17 cells was inhibited, while Treg cells secreted more anti-inflammatory cytokines in centenarians.

## Discussion

Inflammaging is suggested to be one of the major contributory factors leading to the increased morbidity and mortality of older adults; however, the inflammaging status, especially the subsets of CD4^+^ T cells in centenarians is not clearly understood. Herein, it was found that centenarians had unique levels of inflammatory cytokines and reduced Th17/Treg levels. CD4^+^ T cells in centenarians tended to differentiate into pro-inflammatory cells with decreased secretory function. These results suggested the presence of a mechanism in centenarians that alleviated inflammaging. This may be through the reversal of the imbalance of Th17/Treg cells and the reduction of pro-inflammatory cytokines.

Associated with immune dysregulation, inflammaging has been attributed to a combination of age-related defects (Chambers and Akbar, 2020). One of the most evident characteristics of inflammaging is high blood levels of pro-inflammatory mediators, including CRP, TGF-β, TNF-α, IFN-γ, IL-1, and IL-6, in the absence of evident triggers (Ferrucci et al., 2010; Ferrucci and Fabbri, 2018). The levels of these pro-inflammatory mediators have an important relationship with the processes of longevity and aging-related diseases and are positively correlated with mortality (Chung et al., 2009; Ventura et al., 2017; Olivieri et al., 2021). In this study, we detected the levels of inflammation-related factors in the plasma of centenarians and demonstrated that many pro-inflammatory factors, namely, CRP, IL-12, TNF-α, IFN-γ, and IL-6, were elevated in centenarians. Intriguingly, other proinflammatory cytokines, such as IL-17A, IL-1β, and IL-23, were reduced in centenarians (Figure 1). This evidence suggested that centenarians partly alleviated inflammaging by affecting the secretion of these cytokines.

Inflammaging can be partially attributed to dysfunctional or senescent T cells (Mittelbrunn and Kroemer, 2021). Recent evidence suggests that T lymphocytes can directly promote inflammaging through the production of inflammatory cytokines (Desdín-Micó et al., 2020). Metabolic stress in T cells accelerates inflammaging (Lenaers et al., 2020), thus accelerating pathologies that constitute major causes of human frailty and mortality, such as neurodegenerative disorders, chronic kidney disease, metabolic and cardiovascular diseases (Furman et al., 2019). Notably, the presence of a subset of CD4^+^ T cells is associated with elevated circulating inflammatory cytokines (Elyahu et al., 2019). Studies have reported that with aging, CD4 increases and CD8 decreases (Aiello et al., 2019). Paradoxically, Alberro et al., found that senescent CD8 cells accumulate with age, while there is a partial reduction of senescent CD4 cells in nonagenarians and centenarians (Ligotti et al., 2021). In addition, expansion of age-associated cytotoxic CD4^+^ T cells has been identified in human supercentenarians (Hashimoto et al., 2019). However, in our study, CD4^+^ and CD8^+^ T cells were not found to change with age, nor was the CD4/CD8 ratio. In agreement with our findings, Ligotti et al., found a constant trend in the percentages of both CD4^+^ and CD8^+^ T cells with age. Consequently, they did not observe the described age-related increase in the rate of inversion of the CD4/CD8 ratio (Ligotti et al., 2021). Alberro et al. also found no significant differences in CD4/CD8 ratio, despite significant interindividual differences, especially in the old adults (Alberro et al., 2019).

Among CD4^+^ T cells, Th17 cells are unique proinflammatory cells identified by ROR-γt and IL-17 (Mangan et al., 2006). Treg cells are characterized by the expression of both surface CD4^+^ and CD25^+^ and the intracellular transcription factor Foxp3 (Mangan et al., 2006). Tregs promote anti-inflammatory cytokine production (TGF-β and IL-10) and exert a dominant-negative regulatory effect on other Th cells, including Th17 cells (Fantini et al., 2009). An increased Th17/Treg cell ratio was found in patients with autoimmune or inflammatory diseases and plays an important role in the occurrence and development of these diseases (Knochelmann et al., 2018; Zhou et al., 2018; Zhang et al., 2019; Zhang et al., 2020). However, there is still controversy over Th17 and Treg levels changing with age. For instance, Schmitt et al. found that compared with those under 65 years old, there was a significant increase in Th17 cells and a decrease in Tregs in 65 to 80-year-old people (Schmitt et al., 2013). It was also suggested that both Treg cells and Th17 cells increased with age (Van et al., 2014) and that the suppressive activity of Tregs on other cells decreased (Tsaknaridis et al., 2003; Gregg et al., 2005). These differences may be explained by the introduction of biases caused by age and health status differences of the selected population in different studies. In this study, we demonstrated that the level of Th17/Treg cells in centenarians showed an opposite trend with aging; that is, the Th17/Treg ratio decreased compared with that in old adults, which was consistent with the expression level of Th17/Treg-related cytokines (IL-17A, IL-1β, IL-23, and TGF-β), as shown in Figure 1. This evidence indicated that centenarians may reverse the age-related Th17/Treg imbalance (Figure 2). The decreased Th17/Treg ratio may play an important role in alleviating inflammaging and increasing lifespan in centenarians.

*In vitro* T cell cultures from different ages provided controversial results. We found that naive T cells of centenarians tended to differentiate into Th17 cells instead of Tregs, which was demonstrated in previous studies. Studies have shown that naive CD4^+^ T cells from aged animals differentiate into Th17 effectors more readily than T cells from young animals (Huang et al., 2008). This tendency of Th17 polarization seems to be an inherent characteristic of naive CD4^+^ T cells from older individuals. Furthermore, we demonstrated that they secreted fewer proinflammatory cytokines and relatively more anti-inflammatory cytokines (Figure 3 and Figure 4). This was consistent with previous studies (Bektas et al., 2013), and this phenomenon may be associated with altered metabolic activity (Bektas et al., 2014). Previous studies have found that CD4^+^ T cells in centenarians have a senescent pro-inflammatory phenotype (Alberro et al., 2019). This study showed that centenarians had very specific changes in CD4^+^ T cell populations, which were manifested by an elevated Th17/Treg ratio *in vivo*, as well as a changed secretory phenotype. Although the T cells of centenarians cannot resist the aging-related expression of proinflammatory genes, their secretory phenotype was altered, explaining the relatively low level of inflammation in centenarians. These results suggested the presence of a mechanism to ameliorate inflammaging in centenarians. This may be achieved by reversing the imbalance of Th17/Treg cells and reducing pro-inflammatory cytokines.

## Conclusion

Aging is a highly complex process in which inflammaging plays a significant role. Many changes in the immune system with age have been described, most of which are thought to be deleterious and are considered causes of many age-related diseases. In this study, we demonstrated that centenarians alleviated inflammaging by regulating the homeostasis of Th17/Treg cells and related cytokines, which provided novel targets for antiaging drug development. Future research needs to further elucidate the trends described in this study to improve the healthspan and lifespan of older adults.

## Abbreviationsabbreviations

CRP, C-reactive protein; IL, interleukin; TNF-α, tumor necrosis factor-α; IFN-γ, interferon-γ; TGF-β, transforming growth factor-β; Th17, T helper cell 17; Treg, regulatory T cells; ROR-γt, retinoid-related orphan nuclear receptor-γt; Foxp3, forkhead box protein P3, FCM, flow cytometry; PBMCs, peripheral blood mononuclear cells; ELISA, enzyme-linked immunosorbent assay.

## Data Availability

The original contributions presented in the study are included in the article/Supplementary Material, further inquiries can be directed to the corresponding authors.
