# Supplementary material for: Centenarians Alleviate Inflammaging by Changing the Ratio and Secretory Phenotypes of T Helper 17 and Regulatory T Cells
Source: Front Pharmacol. 2022 Jun 2;13:877709. doi: 10.3389/fphar.2022.877709 (PMC9203077; doi:10.3389/fphar.2022.877709)
Supplement: Supplementary file 1 [file DataSheet1.doc]

***Supplementary manuscript***

**Title: Centenarians alleviate inflammaging by changing the ratio and secretory phenotypes of T helper 17 and regulatory T cells**

**Figure Legend**


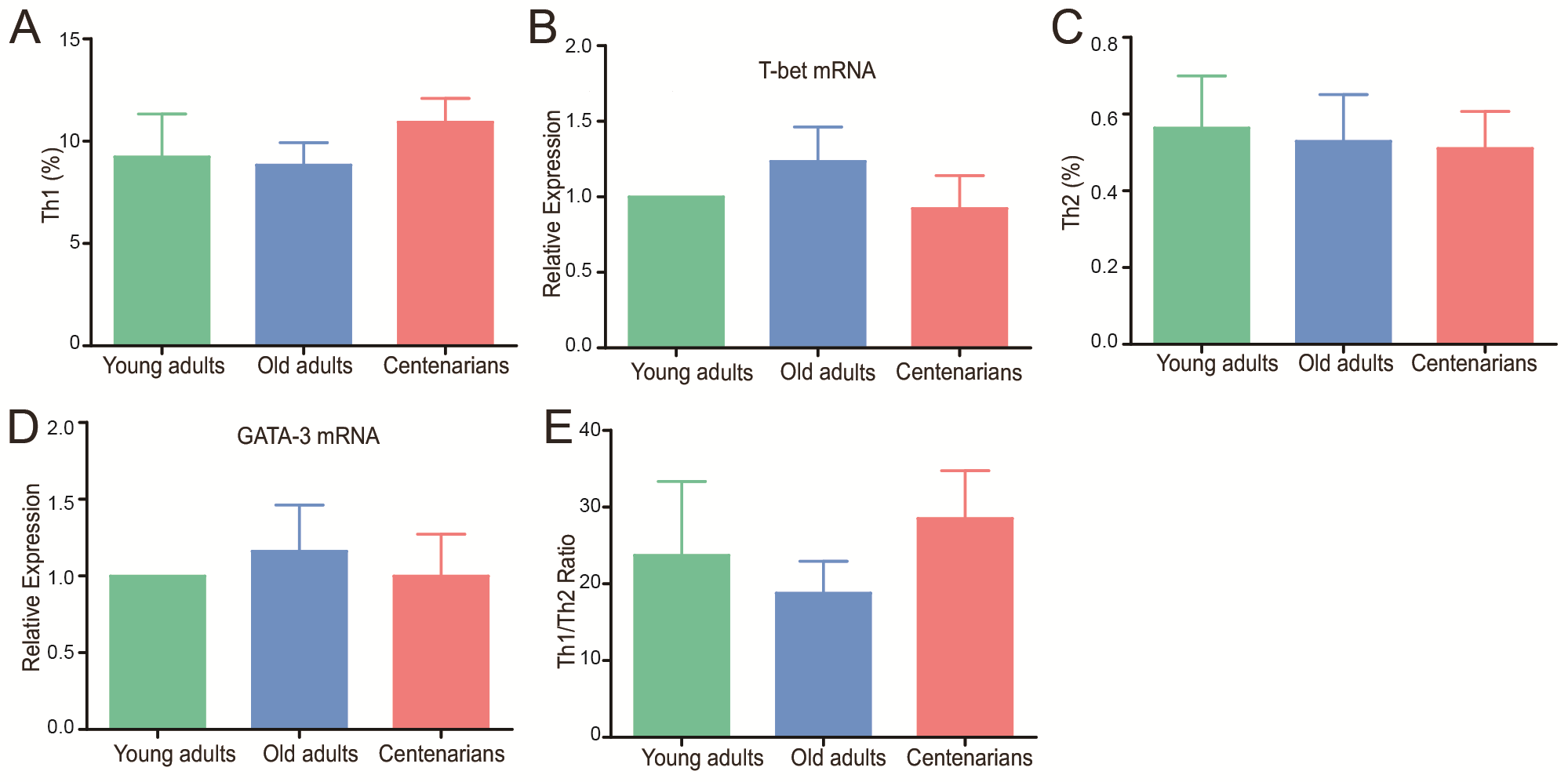


**Supplementary Figure 1. Th1 and Th2 cell ratio in centenarians.**

PBMCs were extracted from young adults, old adults and centenarians. (A) Th1 cells were detected by FCM. (B) The mRNA levels of T-bet in human PBMCs were measured by qPCR. (C) Th2 cells were detected by FCM. (D) The mRNA levels of GATA-3 in human PBMCs were measured by qPCR. (E) The ratio of Th1 to Th2 cells was analyzed. Data were expressed as the mean ± SEM.

**Supplementary Table 1. Characteristics of the study centenarians.**

| Characteristics | Male | Female | Total | P value |
| --- | --- | --- | --- | --- |
| N (%) | 55 (25.2) | 163 (74.8) | 218 (100.0) | 0.001^*^ |
| Age (χ±SD) | 102.3±1.7 | 102.2±2.1 | 102.2±2.0 | 0.9007 |
| Current smokers (N,%) | 12 (21.8) | 4 (2.5) | 16 (7.3) | <0.001^*^ |
| Chronic diseases (N,%) |  |  |  |  |
| Hypertension | 6 (10.9) | 27 (16.6) | 33 (15.4) | 0.3116 |
| Myocardial disease | 4 (7.3) | 13 (8.0) | 17 (7.8) | 0.8665 |
| Diabetes mellitus | 2 (3.6) | 4 (2.5) | 6 (2.8) | 0.6430 |
| Cerebrovascular disease | 4 (7.3) | 5 (3.1) | 9 (4.1) | 0.1753 |
| Parkinson's disease | 1 (1.8) | 0 (0) | 1 (0.5) | 0.0844 |
| Alzheimer's disease | 6 (10.9) | 17 (10.4) | 23 (10.6) | 0.9202 |
| Respiratory disease | 7 (12.7) | 13 (8.0) | 20 (9.2) | 0.2911 |
| CKD | 4 (7.3) | 0 (0) | 4 (1.8) | <0.001^*^ |
| Tumor of any type | 2 (3.6) | 4 (2.5) | 6 (2.8) | 0.6430 |
| Osteoarthritis | 1 (1.8) | 6 (3.7) | 7 (3.2) | 0.4980 |
| ADL score (χ±SD) | 80.4±20.4 | 76.6±22.8 | 77.6±22.3 | 0.2827 |
| Falls in the previous 12 months (χ±SD） | 1.1±1.3 | 1.7±2.4 | 1.6±2.2 | 0.0801 |
| BMI (kg/m^2^,χ±SD) | 20.6±3.7 | 22.2±7.5 | 21.8±6.8 | 0.1237 |
| Systolic pressures (mmHg,χ±SD) | 140.3±16.3 | 145.7±22.7 | 144.4±21.4 | 0.1301 |
| Diastolic pressures (mmHg,χ±SD) | 80.1±14.1 | 82.5±15.0 | 81.9±14.8 | 0.3236 |
| Heart rate (χ±SD) | 61.7±27.9 | 61.0±31.8 | 61.1±30.8 | 0.8817 |

Groups were compared by nonparametric analyses.

BMI, body mass index; CKD, Chronic kidney disease

*: *P*<0.05.

**Supplementary Table 2. Routine blood test of the study centenarians.**

| Items | Male | Female | Total | *P* value |
| --- | --- | --- | --- | --- |
| WBC (×10^9^/L) | 5.826±1.645 | 5.899±1.939 | 5.881±1.868 | 0.2194 |
| RBC (×10^12^/L) | 3.947±0.8049 | 3.938±0.4901 | 3.94±0.5782 | 0.8021 |
| HGB (g/L) | 120.5±20.23 | 114.3±13.91 | 115.8±15.81 | 0.0578 |
| PLT (×10^9^/L) | 156.0±73.53 | 181.0±66.18 | 175.0±68.62 | 0.0197***** |
| GRAN (%) | 54.97±9.936 | 58.27±9.817 | 57.48±9.916 | 0.0771 |
| LYM (%) | 30.87±10.19 | 30.27±9.332 | 30.41±9.514 | 0.5178 |
| MONO (%) | 9.372±3.558 | 8.288±2.780 | 8.547±3.009 | 0.1061 |
| EOS (%) | 4.185±3.813 | 2.663±2.259 | 3.027±2.776 | 0.0545 |
| BAS (%) | 0.6000±0.3187 | 0.5081±0.2965 | 0.5301±0.3035 | 0.7398 |

Data are presented as the mean ± standard deviation (SD). WBC: white blood cell count; RBC: red blood cell; HGB: hemoglobin; PLT: platelet count; GRAN: granulocyte; LYM: lymphocyte; MONO: monocyte; EOS: eosinophil; BAS: basophil.*: *P*<0.05.

**Supplementary Table 3. Metabolic characteristics of study centenarians.**

| Items | Male | Female | Total | *P* value |
| --- | --- | --- | --- | --- |
| GLU (mmol/L) | 6.464±2.185 | 6.251±1.935 | 6.302±1.993 | 0.8918 |
| CHOL (mmol/L) | 4.267±0.9262 | 4.534±0.8630 | 4.470±0.8830 | 0.4407 |
| TG (mmol/L) | 1.248±0.6562 | 1.398±0.8221 | 1.362±0.7863 | 0.7064 |
| HDL (mmol/L) | 1.509±0.3417 | 1.668±0.4124 | 1.630±0.4014 | 0.4834 |
| LDL (mmol/L) | 2.135±0.8137 | 2.176±0.6187 | 2.166±0.6680 | 0.665 |
| TP (g/L) | 69.52±6.677 | 69.25±5.853 | 69.31±6.040 | 0.3841 |
| ALB (g/L) | 40.01±3.876 | 40.81±3.676 | 40.62±3.729 | 0.7476 |
| GLOB (g/L) | 29.29±5.038 | 28.32±4.892 | 28.57±4.935 | 0.9791 |
| A/G | 1.395±0.2679 | 1.471±0.2669 | 1.453±0.2684 | 0.4733 |
| PA (g/L) | 151.1±40.67 | 167.4±38.09 | 163.5±39.21 | 0.0068****** |
| TB (μmol/L) | 8.123±3.642 | 7.206±3.874 | 7.426±3.829 | 0.1168 |
| DB (μmol/L) | 3.528±1.497 | 3.14±1.487 | 3.233±1.494 | 0.2682 |
| IB (μmol/L) | 4.595±2.652 | 4.066±2.664 | 4.193±2.663 | 0.1191 |
| eGFR (mL/min) | 39.02±13.58 | 45.7±13.91 | 43.98±14.10 | 0.0011****** |
| CREA (mmol/L) | 100.9±32.26 | 76.00±26.21 | 81.96±29.66 | 0.0003******* |
| UA (mmol/L) | 389.3±95.60 | 336.8±95.12 | 349.4±97.56 | 0.0204***** |
| Urea (mmol/L) | 8.755±2.947 | 7.187±2.233 | 7.563±2.505 | 0.0244***** |
| ALT (U/L) | 15.49±9.228 | 12.27±7.372 | 13.04±7.945 | 0.3727 |
| AST (U/L) | 25.58±8.120 | 23.07±8.788 | 23.72±8.670 | 0.3909 |
| ALP (U/L) | 88.09±27.51 | 93.8±34.47 | 92.32±32.83 | 0.9138 |
| GGT (U/L) | 27.92±24.27 | 19.9±19.55 | 21.99±21.11 | 0.307 |
| LDH（U/L） | 221.7±60.60 | 228.3±56.94 | 226.6±57.83 | 0.4169 |
| TBA (μmol/L) | 14.79±11.02 | 14.2±12.70 | 14.35±12.27 | 0.598 |
| Na (mmol/L) | 140.8±1.913 | 141±2.604 | 140.9±2.442 | 0.3091 |
| Cl (mmol/L) | 103.7±3.626 | 103.4±4.095 | 103.4±3.974 | 0.2931 |
| K (mmol/L) | 4.205±0.6396 | 4.440±0.8566 | 4.384±0.8144 | 0.0489***** |
| Mg (mmol/L) | 0.8092±0.06313 | 0.8323±0.07953 | 0.8268±0.07638 | 0.1059 |
| P (mmol/L) | 25.81±7.286 | 26.41±7.272 | 26.25±7.262 | 0.2574 |

Data are presented as the mean ± standard deviation (SD). GLU: glucose; CHOL: cholesterol; TG: triglyceride; HDL, high-density lipoprotein; LDL, low-density lipoprotein; TP: total protein; ALB: albumin; GLOB: globulin; A/G: albumin/globulin; PA: prealbumin; TB: total bilirubin; DB: direct bilirubin; IB: indirect bilirubin; eGFR: estimated glomerular filtration rate; CREA, creatinine; UA: uric acid; ALT, alanine transaminase; AST, aspartate aminotransferase; ALP: alkaline phosphatase; GGT: γ-glutamyl transpeptidase; LDH: lactate dehydrogenase; TBA: total biliary acids; Na: natrium; Cl: chlorine; K: kalium; Mg: magnesium; P: phosphorus.*: *P*<0.05; **: *P*<0.01; ***: *P*<0.005.
